# Olfactory function is longitudinally associated with semantic fluency in Parkinson’s disease: a cohort study

**DOI:** 10.1007/s00415-025-13337-0

**Published:** 2025-09-02

**Authors:** Dareia S. Roos, Henk W. Berendse, Richard L. Doty, Jos W. R. Twisk, Martin Klein

**Affiliations:** 1 Department of Neurology, Amsterdam UMC Location Vrije Universiteit Amsterdam, de Boelelaan 1117, 1081 HV Amsterdam, The Netherlands; 2 Amsterdam Neuroscience, Neurodegeneration, Amsterdam, The Netherlands; 3 Department of Medical Psychology, Amsterdam UMC Location Vrije Universiteit Amsterdam, de Boelelaan 1117, 1081 HV Amsterdam, The Netherlands; 4 Perelman School of Medicine, University of Pennsylvania, Philadelphia, USA; 5 Smell and Taste Evaluation Center, Haddon Heights, NJ USA; 6 Department of Epidemiology and Data Science, Amsterdam UMC Location Vrije Universiteit Amsterdam, de Boelelaan 1117, 1081 HV Amsterdam, The Netherlands

**Keywords:** Parkinson’s disease, Olfaction, Cognitive functioning

## Abstract

**Background:**

Olfactory dysfunction is an early, common, and progressive symptom in Parkinson’s disease (PD). Whether the decline in olfactory function is longitudinally associated with a deterioration of (non-)motor symptoms remains debated.

**Objectives:**

This study aimed to investigate the longitudinal relationship between olfactory function and (non-)motor symptoms, particularly cognitive decline, in PD patients over a ten-year follow-up period.

**Methods:**

Ninety patients were assessed at baseline and after approximately ten years. Olfactory function was measured using the 40-item University of Pennsylvania Smell Identification Test (UPSIT®). (non-)Motor symptoms were evaluated using various scales and questionnaires, including the MMSE to assess global cognitive function. Linear regression was used to analyze the change in olfactory function over time in relation to changes in (non-)motor function, and to determine whether baseline olfactory test scores would be associated with (non-)motor function at follow-up. In a subset of 62 patients, in whom comprehensive cognitive testing was performed, we analyzed the longitudinal relationship between olfactory function and performance on specific cognitive tests.

**Results:**

Statistically significant associations were found between a decrease in UPSIT® scores and decline in MMSE, and between baseline UPSIT® scores and MMSE performance at follow-up. In the subgroup with comprehensive cognitive testing, a decrease in UPSIT® scores was associated with worsening semantic fluency. Furthermore, an association was found between baseline UPSIT® score and semantic fluency at follow-up.

**Conclusions:**

Decline in olfactory function in PD is longitudinally associated with worsening global cognitive function, particularly a deterioration in semantic fluency. Baseline olfactory function may be predictive of later cognitive decline, especially in the semantic domain.

**Supplementary Information:**

The online version contains supplementary material available at 10.1007/s00415-025-13337-0.

## Introduction

With a prevalence of up to 90%, olfactory dysfunction is very common in patients with Parkinson’s disease (PD) [1–3]. Olfactory loss may precede the first disease-related motor symptoms by several years and is considered an early hallmark of the disease [4, 5]. However, the relationship between olfactory dysfunction and other motor and non-motor symptoms in PD remains incompletely understood.

Previous cross-sectional research investigating the association between olfactory function and motor symptoms have yielded inconsistent results. While some studies have reported that the degree of hyposmia correlates with disease severity, as measured with the Unified PD Rating Scale motor subscale (UPDRS III) [6–10], others have found no significant association with disease stage or severity [3, 11–13].

Similarly, studies exploring the relationship between olfactory function and neuropsychiatric symptoms in PD have produced conflicting findings. In one cross-sectional study, neuropsychiatric symptoms, except mood, were more common in patients with poor olfactory function [14]. Conversely, two other studies failed to identify a significant correlation between olfactory dysfunction and psychiatric symptoms in PD [15, 16]. Other studies have suggested links between olfactory dysfunction and other non-motor symptoms, such as REM sleep behavior disorder [17], constipation [17], apathy,[18, 19], and autonomic failure [20]. Poor olfactory function has also been associated with cognitive impairment [14, 21–23]. In a previous baseline analysis of the present cohort, we found significant associations between olfactory dysfunction and motor as well as non-motor symptoms, including cognitive decline, depression, anxiety, autonomic dysfunction, and sleep disturbances [24].

The cross-sectional nature of the aforementioned studies limits the ability to infer causality or temporal relationships between hyposmia and other motor and non-motor symptoms in PD. Longitudinal studies are crucial for understanding in what way olfactory dysfunction evolves over time and how it relates to the progression of motor and non-motor symptoms. While olfactory function is known to decline as PD progresses, its temporal association with other symptoms remains unclear [25, 26]. Doty et al., for instance, observed no significant relationship between olfaction and disease stage or motor symptom severity over a 24-month follow-up period [1]. In contrast, He et al. reported that hyposmic PD patients experienced more severe motor symptoms, required higher doses of dopaminergic medications, and showed poorer global cognition [27], while a five-year study by Martinez-Nunez et al. found no association between olfactory dysfunction and apathy [28]. A few studies have examined whether olfactory function at baseline would predict cognitive functioning over time. In most of these studies hyposmia was associated with an increased risk of dementia [29–31].

These inconsistencies highlight the need for further investigation. Understanding which motor and non-motor symptoms are longitudinally associated with progressive olfactory decline may offer critical insights into the pathophysiological processes underlying PD. According to Braak’s staging hypothesis, alpha-synuclein pathology begins in the olfactory bulb, olfactory nucleus, and lower brainstem before spreading to cortical regions [32]. This pattern suggests that as PD progresses, olfactory dysfunction and other symptoms may worsen concurrently.

The objective of this study was to assess the longitudinal relationship between changes in olfactory function and changes in various motor and non-motor symptoms in PD over a ten-year follow-up period. In addition, we wanted to assess whether baseline olfactory function would be associated with motor and non-motor symptom severity ten years from baseline. By doing so, we aimed to deepen our understanding of the interplay between these symptoms and the pathological changes that occur over time in PD.

## Methods

### Patients

The baseline data for the present study were collected as part of routine clinical care in a cohort consisting of 295 PD patients who were recruited from the outpatient clinic for movement disorders at Amsterdam UMC, location Vrije Universiteit Amsterdam, between May 2008 and February 2014, as described in more detail elsewhere [24]. The follow-up data for the study were obtained after an average interval of approximately ten years. A total of 90 patients, with a mean age of 58 years, could be included. All other patients in the original cohort of 295 PD patients were either deceased (45%; *n* = 133), declined to participate (22%; *n* = 65), or were otherwise lost to follow-up (2%; *n* = 7). Table 1 in the supplement shows the baseline characteristics of the patients included in the follow-up study compared to the patients that were lost to follow-up or declined to participate.

**Table 1:** Characteristics of the study population group

	Baseline			Follow-up		
	*N*	Mean (SD)	Range	*N*	Mean (SD)	Range
Age, y		58.3 (9.4)	[27–74]		68.0 (9.4)	[37–84]
Males	62	68.9%				
Disease duration, y		4.5 (5.2)	[0–34]		14.2 (5.4)	[8–44]
LEDD, mg		227.4 (352)	[0–1255]		985.4 (504)	[200–2265]
UPSIT® score		22.2 (7.6)	[3–36]		16.0 (5.5)	[6–33]
H&Y stage						
Stage 1	17	18.9%		–	–	
Stage 1.5	12	13.3%		–	–	
Stage 2	46	51.1%		29	32.2%	
Stage 2.5	10	11.1%		27	30.0%	
Stage 3	5	5.6%		24	26.7%	
Stage 4	–	–		9	10.0%	
Stage 5	–	–		1	1.1%	
UPDRS III	88	21.7 (10.6)	[4–46]	89	25.6 (11.9)	[6–66]
MMSE	88	28.6 (1.5)	[23–30]	87	27.1 (3.6)	[8–30]
SCOPA-SLEEP	84	11.1 (7.9)	[2–35]	89	12.8 (7.8)	[1–37]
SCOPA-AUT	86	11.0 (7.2)	[0–33]	88	16.1 (7.5)	[1–37]
SCOPA-PC	87	0.8 (1.1)	[0–5]	89	1.8 (1.9)	[0–9]
BAI	87	11.9 (9.2)	[0–41]	89	12.7 (11.3)	[0–37]
BDI	89	8.8 (7.4)	[0–44]	89	7.4 (5.8)	[0–38]

*LEDD* levodopa equivalent daily dosage; *H&Y* Hoehn & Yahr; *UPDRS III* Unified Parkinson’s Disease Rating Scale motor subscale; *MMSE* Mini-Mental State Examination; *SCOPA* SCales for Outcomes in PArkinson’s Disease; *AUT* autonomic dysfunction; *PC*: Psychiatric complications; *BAI* Beck Anxiety Inventory; *BDI* Beck Depression Inventory

At the follow-up visit, all of the follow-up study patients still fulfilled the UK Parkinson’s Disease Brain Bank criteria for the clinical diagnosis of PD [33], as determined by a neurologist specialized in movement disorders (DR). Follow-up visits were performed between August 2020 and June 2021, either at the patient’s home (*n* = 44) or the outpatient clinic (*n* = 46), depending on their individual preferences. This study was approved by the Medical Ethical Committee of Amsterdam UMC, location Vrije Universiteit Amsterdam (VUmc; 2020.012). All patients gave written informed consent.

### Olfactory testing

The Dutch version of the University of Pennsylvania Smell Identification Test (UPSIT®) was employed both at baseline and follow-up [34]. This highly reliable self-administered forced-choice 40-item odor identification test (test–retest *r* = 0.92) has been translated into over 60 languages including Dutch and is strongly correlated with odor detection threshold tests (e.g., phenyl ethyl alcohol *r* = 0.89 [34]; n-butanol *r* = 0.92) [35]. After scratching a patch containing microcapsules filled with odorant, the subject sniffs the released odorant, and has to choose one out of four response options. The total score is calculated by adding up all correct answers. At baseline, patients completed the UPSIT® at home. When the UPSIT® was incomplete, subjects were asked to fill in the missing items at the hospital. At follow-up, UPSIT® testing was supervised and, when necessary, the researcher helped scratching the patch. In only one case, a single item was missing due to a manufacturing error; the average of the non-missing values was used to impute the missing value.

### Clinical profiling

#### Motor symptoms

Clinical measures of disease severity were determined at baseline and follow-up. To assess motor symptom severity, we used the UPDRS III (range 0–108) [36]. Patients were on medication when rated. Disease stage was defined using the modified Hoehn & Yahr stage (H&Y) [37]. Disease duration in years was measured from the onset of subjective motor symptoms.

To calculate the levodopa equivalent daily dose (LEDD) the total dose of dopaminergic medication was converted using the following conversion rate: 100 mg levodopa equaling 133.33 mg levodopa with controlled release, 1 mg pramipexol (as salt), 5 mg ropinirole, 3.3 mg rotigotine, and 100 mg amantadine. Additionally, 10% was added to the total levodopa dose in case of the use of selegiline, rasagiline or safinamide, while 20% was added to the total levodopa dose in case of the use of a catechol-O-methyl transferase (COMT) inhibitor [38].

#### Cognitive function

Global cognitive function was tested using the Mini-Mental State Examination (MMSE)[39] at baseline and follow-up. At baseline, comprehensive cognitive testing was performed in a subgroup of patients for whom this was deemed clinically necessary, as per the referring physician. At follow-up, comprehensive cognitive testing was performed in 62 patients. As a result, since not all patients performed comprehensive cognitive testing at baseline, test outcomes of 52 patients on the following tests were available both at baseline and follow-up: attention and working memory (Digit Span forward and backward[40] and Stroop Color-Word test[41]), executive function (phonemic fluency[42] and semantic fluency[42]), and verbal memory (Rey’s Auditory Verbal Learning Test delayed recall [RAVLT][43]). The level of education was classified using the system of Verhage, ranging from level 1 (elementary school not finished) to level 7 (university) [44].

#### Other non-motor symptoms

To assess autonomic symptoms the SCales for Outcomes in PArkinson’s disease Autonomic dysfunction (SCOPA-AUT) was used [45]. Sleep disturbances were assessed using the SCales for Outcomes in PArkinson’s disease for sleep (SCOPA-SLEEP) [46]. The SCales for Outcomes in PArkinson’s disease Psychiatric Complication (SCOPA-PC) was used to evaluate psychiatric symptoms [47]. We rated depressive symptoms and anxiety symptoms using the Beck Depression Inventory (BDI) [48] and the Beck Anxiety Inventory (BAI) [49], respectively. All tests of non-motor function were performed at baseline and follow-up. For all tests except cognitive testing, a higher score reflects more severe symptoms.

### Statistical analysis

The data were analyzed using SPSS version 28.0 (SPSS Inc., Chicago, IL, USA). The significance level.

was set at 0.05.

The baseline demographic characteristics of the PD patients that were included in the follow-up study and the patients that could not be included were compared using an independent *t*-test, Mann–Whitney *U* test, or Chi-square test, depending on the distribution of the data (supplement Table 1). The same tests were used to compare the demographics of the group of patients with comprehensive cognitive testing versus the participants without these additional tests (supplement Table 2).

The mean scores at baseline and follow-up for the various motor, non-motor, and comprehensive cognitive tests were calculated. Additionally, we examined whether the differences between baseline and follow-up were statistically significant.

To analyze the longitudinal relationship between change in olfactory test scores and the changes in motor and other non-motor symptoms, a linear regression analysis was used. For each variable, three analyses were performed: the first uncorrected (model 1), the second adjusted for age and sex (model 2), and the third further adjusted for disease duration (model 3).

In the subgroup of patients in whom comprehensive cognitive testing had been performed, we additionally tested the longitudinal relationship between change in olfactory test scores and change in the different specific cognitive test outcomes. Neuropsychological test results were first transformed to t-scores, which included a correction for educational level and, depending on the test psychometrics, also a correction for age and/or sex.

To analyze the relationship between baseline olfactory function and the various motor and non-motor variables at follow-up, a linear regression analysis was used. For each variable, four analyses were performed: the first three according to the method described above, with the addition of a fourth model in which an additional adjustment for the interval between baseline and follow-up visit was used. Data from the SCOPA-SLEEP and the BDI were first log transformed because of non-normally distributed residuals. Lastly, in the subgroup of patients in whom comprehensive cognitive testing had been performed, we also used a linear regression with the same models described above to analyze whether baseline olfactory test scores would be associated with the different specific cognitive test scores at follow-up. This group consisted of 62 patients.

## Results

### Clinical characteristics

The mean age of the patients at follow-up was 68 years, with a mean disease duration of 14 years. As expected, the LEDD was higher at follow-up than at baseline (Table 1). The subgroup of patients who underwent comprehensive cognitive testing at follow-up were younger at baseline, were more often male, had a higher baseline MMSE score, and had a similar disease duration and LEDD compared to patients who did not undergo comprehensive cognitive testing at follow-up (Supplement Table 2).

**Table 2:** Results of the linear regression analyses relating change in UPSIT® score to change in motor and non-motor variables

	N		β	B (95% CI)	Sign. (*p*)
UPDRS III	87	Model 1	0.071	0.152 (−0.309; 0.614)	0.513
		Model 2	0.090	0.194 (−0.271; 0.658)	0.410
		Model 3	0.090	0.193 (−0.274; 0.661)	0.413
MMSE	85	Model 1	−0.204	−0.114 (−0.233; 0.005)	0.061
		Model 2	−0.226	−0.126 (−0.247; −0.004)	0.043*
		Model 3	−0.226	−0.126 (−0.247; −0.005)	0.042*
SCOPA-SLEEP	82	Model 1	0.092	0.145 (−0.206; 0.496)	0.413
		Model 2	0.088	0.140 (−0.216; 0.495)	0.437
		Model 3	0.088	0.139 (−0.218; 0.496)	0.441
SCOPA-AUT	84	Model 1	−0.050	−0.065 (−0.353; 0.223)	0.653
		Model 2	−0.026	−0.034 (−0.324; 0.256)	0.815
		Model 3	−0.026	−0.035 (−0.327; 0.257)	0.814
SCOPA-PC	86	Model 1	−0.106	−0.036 (−0.109; 0.037)	0.330
		Model 2	−0.100	−0.034 (−0.106; 0.039)	0.356
		Model 3	−0.102	−0.034 (−0.107; 0.039)	0.352
BAI	86	Model 1	−0.137	−0.311 (−0.798; 0.177)	0.209
		Model 2	−0.124	−0.281 (−0.782; 0.221)	0.269
		Model 3	−0.109	−0.248 (−0.714; 0.217)	0.292
BDI	88	Model 1	−0.017	−0.020 (−0.274; 0.235)	0.877
		Model 2	−0.007	−0.008 (−0.272; 0.255)	0.950
		Model 3	−0.007	−0.008 (−0.273; 0.257)	0.954

Model 1 for UPSIT® only

Model 2: adjusted for sex and age

Model 3: as model 2 with adjustment for disease duration

Β standardized coefficient; B unstandardized coefficient; *CI*confidence interval

*Significant (*p* < 0.05)

*UPDRS III* Unified Parkinson’s Disease Rating Scale motor subscale, *MMSE* Mini-Mental State Examination, *SCOPA* SCales for Outcomes in PArkinson’s Disease, *AUT* Autonomic dysfunction, *PC* Psychiatric complications, *BAI* Beck Anxiety Inventory, *BDI* Beck Depression Inventory

### Relationship between change in olfactory function and change in (non-)motor symptoms

Between baseline and follow-up, most measures of motor and non-motor symptoms showed an increase in symptom severity, except BDI and both forward and reverse digit span tests (Table 1, supplement Table 3).

**Table 3:** Results of the linear regression analyses relating change in UPSIT® score to change in test scores on specific cognitive tests

	N		β	B (95% CI)	Sign. (*p*)
Rey’s Auditory Verbal Learning Test	51	Model 1	−0.138	−0.281 (−0.862; 0.299)	0.335
delayed recall		Model 2	−0.025	−0.052 (−0.617; 0.513)	0.855
		Model 3	−0.025	−0.052 (−0.623; 0.520)	0.857
Stroop Color-Word test	50	Model 1	−0.057	−0.092 (−0.563; 0.379)	0.696
		Model 2	0.074	0.121 (−0.346; 0.587)	0.606
		Model 3	0.074	0.120 (−0.350; 0.591)	0.609
Forward Digit Span	52	Model 1	−0.156	−0.369 (−1.035; 0.296)	0.270
		Model 2	−0.172	−0.408 (−1.103; 0.287)	0.244
		Model 3	−0.175	−0.414 (−1.096; 0.268)	0.228
Reverse Digit Span	52	Model 1	0.004	0.008 (−0.560; 0.575)	0.978
		Model 2	0.049	0.098 (−0.496; 0.692)	0.742
		Model 3	0.049	0.099 (−0.501; 0.698)	0.742
Phonemic fluency	50	Model 1	0.017	0.031 (−0.484; 0.545)	0.906
		Model 2	−0.101	−0.178 (−0.685; 0.328)	0.482
		Model 3	−0.098	−0.174 (−0.675; 0.328)	0.489
Semantic fluency	50	Model 1	−0.291	−0.600 (−1.172; −0.027)	0.040*
		Model 2	−0.371	−0.764 (−1.298; −0.230)	0.006*
		Model 3	−0.369	−0.761 (−1.299; −0.223)	0.007*

Model 1 for UPSIT® only

Model 2: adjusted for sex and age

Model 3: as model 2 with adjustment for disease duration

Β standardized coefficient; B unstandardized coefficient; *CI* confidence interval.

*Significant (*p* < 0.05)

The decline in UPSIT® test scores over the follow-up period was significantly associated with a decline in MMSE score in both model 2, correcting for sex and age, and model 3, that corrected also for disease duration. The association did not survive a Bonferroni correction for multiple comparisons. There were no statistically significant relationships between change in UPSIT® scores and changes in any of the other motor or non-motor variables (Table 2).

**Fig. 1:**
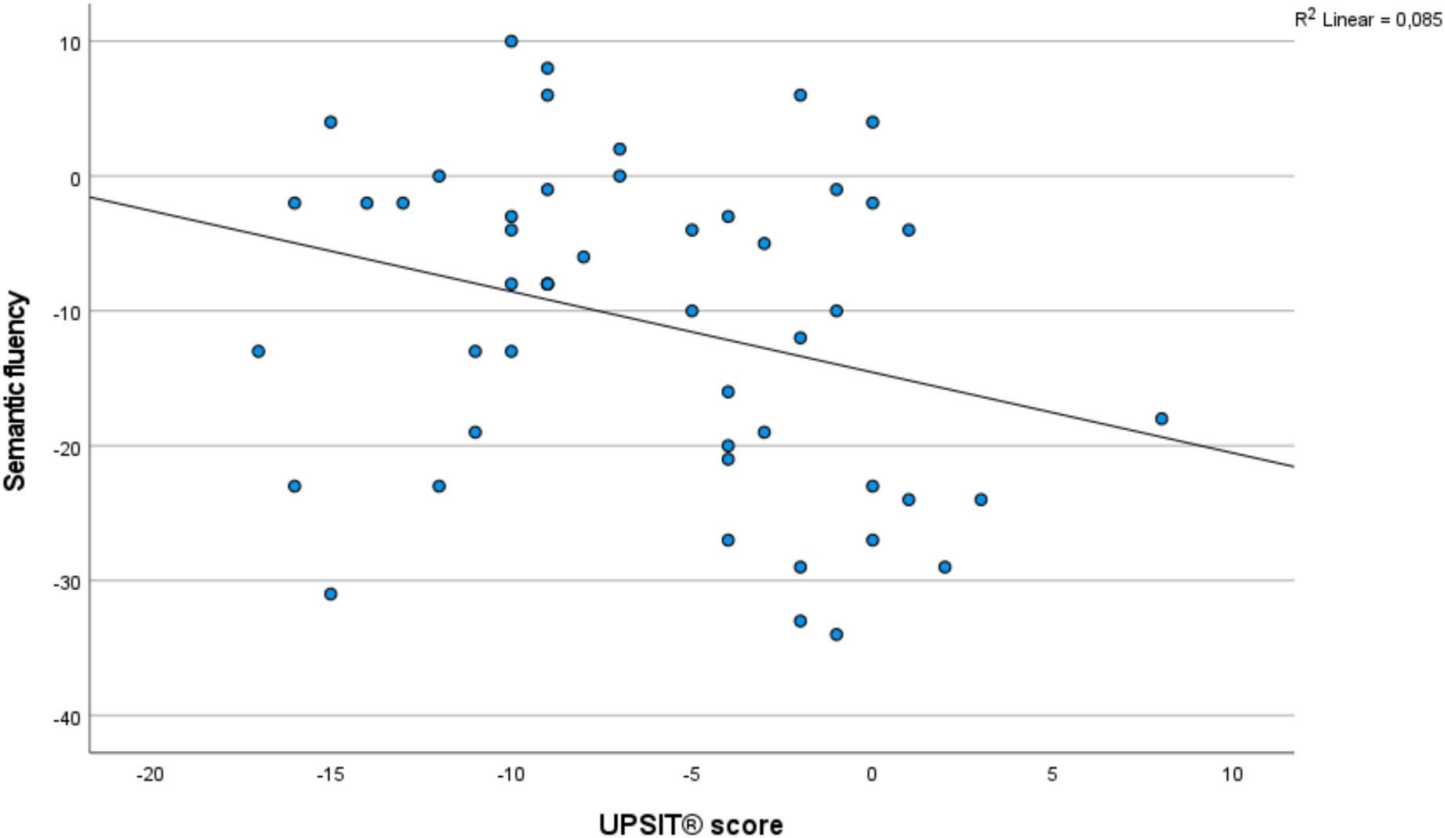
Scatterplot showing the relation between change in UPSIT® score and change in semantic fluency

In the subgroup of patients in whom comprehensive cognitive testing had been performed both at baseline and follow-up (*n* = 52), the decrease in UPSIT® scores associated significantly with a decline in semantic fluency, in all models (Table 3, Fig. 1), and also after applying a Bonferroni correction. There were no significant correlations with any of the other cognitive outcome measures.

### Relationship between baseline olfactory function and severity of (non-)motor symptoms ten years from baseline

Baseline olfactory function was significantly associated with MMSE score and autonomic symptom severity at follow-up in all models at follow-up (Table 4). Furthermore, an association was found between baseline olfactory test score and UPDRS III and SCOPA-PC scores ten years from baseline in model 4. When applying a Bonferroni correction, only the association between baseline olfactory test scores and MMSE remained significant.

**Table 4:** Results of the linear regression analyses relating baseline UPSIT® score to various motor and non-motor variables at follow-up

	N		β	B (95% CI)	Sign. (*p*)
UPDRS III	89	Model 1	−0.415	−0.644 (−0.945; −0.343)	< 0.001*
		Model 2	−0.233	−0.361 (−0.688; −0.034)	0.031*
		Model 3	−0.210	−0.325 (−0.658; 0.007)	0.055
		Model 4	−0.244	−0.379 (−0.717; −0.041)	0.028*
MMSE	87	Model 1	0.463	0.216 (0.127; 0.305)	< 0.001*
		Model 2	0.414	0.193 (0.091; 0.295)	< 0.001*
		Model 3	0.428	0.199 (0.095; 0.303)	< 0.001*
		Model 4	0.481	0.224 (0.121; 0.328)	< 0.001*
SCOPA-SLEEP^a^	89	Model 1	−0.331	−0.014 (−0.022; −0.005)	0.002*
		Model 2	−0.202	−0.008 (−0.018; 0.001)	0.081
		Model 3	−0.202	−0.008 (−0.018; 0.001)	0.087
		Model 4	−0.210	−0.009 (−0.019; 0.001)	0.084
SCOPA-AUT	88	Model 1	−0.425	−0.421 (−0.613; −0.229)	< 0.001*
		Model 2	−0.308	−0.305 (−0.516; −0.095)	0.005*
		Model 3	−0.280	−0.277 (−0.491; −0.063)	0.012*
		Model 4	−0.273	−0.270 (−0.491; −0.049)	0.017*
SCOPA-PC	89	Model 1	−0.290	−0.073 (−0.124; −0.022)	0.006*
		Model 2	−0.193	−0.048 (−0.106; 0.010)	0.104
		Model 3	−0.196	−0.049 (−0.109; 0.011)	0.106
		Model 4	−0.248	−0.062 (−0.122; −0.002)	0.043*
BAI	89	Model 1	0.082	0.121 (−0.190; 0.432)	0.442
		Model 2	0.111	0.163 (−0.193; 0.519)	0.365
		Model 3	0.108	0.159 (−0.206; 0.523)	0.390
		Model 4	0.115	0.168 (−0.207; 0.543)	0.376
BDI^a^	89	Model 1	−0.223	−0.009 (−0.017; −0.001)	0.036*
		Model 2	−0.177	−0.007 (−0.016; 0.002)	0.133
		Model 3	−0.190	−0.008 (−0.017; 0.002)	0.116
		Model 4	−0.157	−0.006 (−0.016; 0.003)	0.201

Model 1 for UPSIT® only

Model 2: adjusted for sex and age

Model 3: as model 2 with adjustment for disease duration

Model 4: as model 3 with adjustment for time between follow-up and baseline

β standardized coefficient; B unstandardized coefficient; *CI* confidence interval

^a^Log transformed data

*Significant (*p* < 0.05)

*UPDRS III* Unified Parkinson’s Disease Rating Scale motor subscale, *MMSE* Mini-Mental State Examination, *SCOPA* SCales for Outcomes in PArkinson’s Disease, *AUT* autonomic dysfunction, *PC* psychiatric complications, *BAI* Beck Anxiety Inventory, *BDI* Beck Depression Inventory

**Fig. 2:**
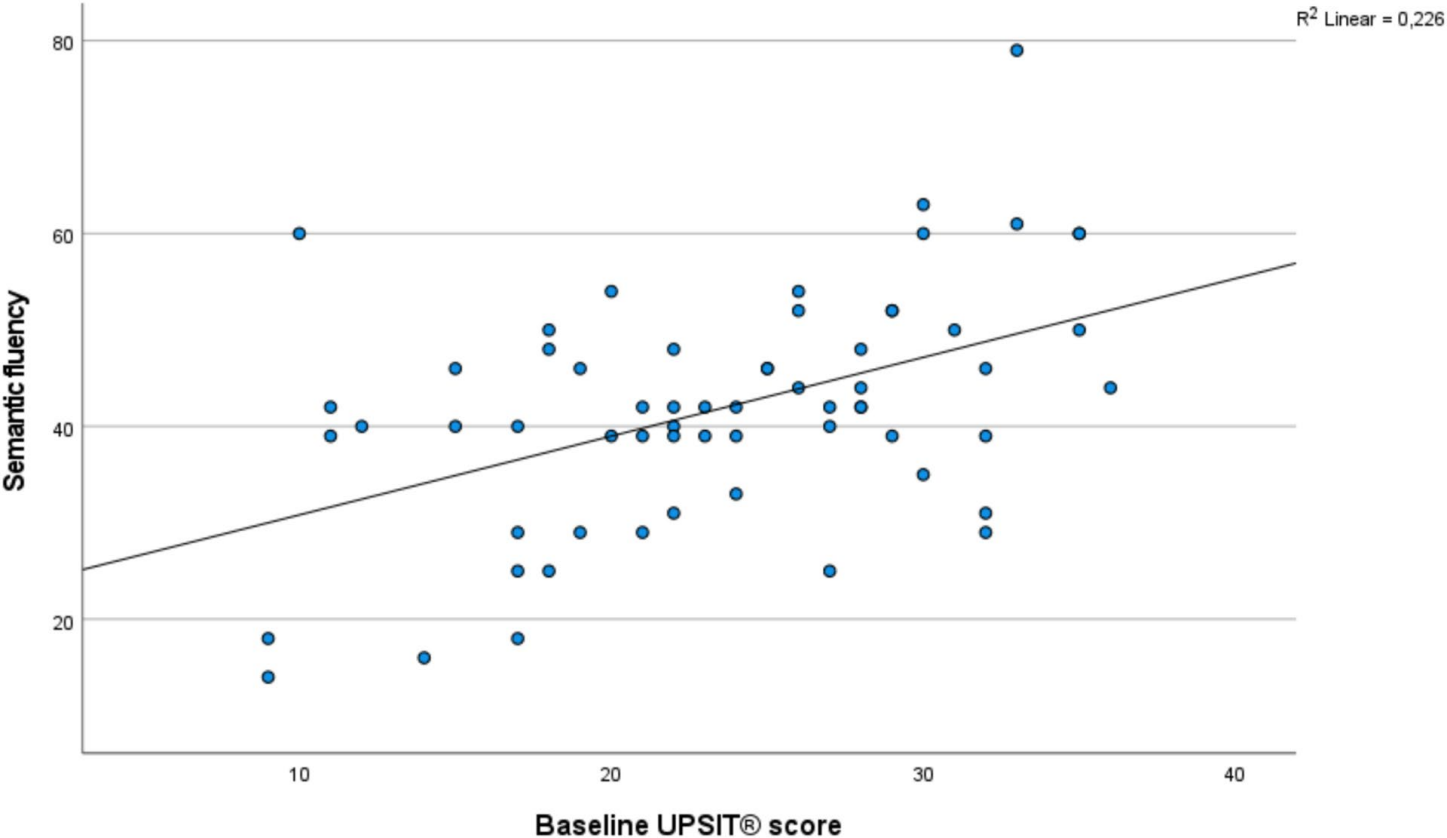
Scatterplot showing the relation between baseline UPSIT® score and semantic fluency at follow-up

In the subgroup of patients with comprehensive cognitive testing at follow-up (*n* = 62), an association was found between baseline olfactory function and semantic fluency at follow-up (Table 5, Fig. 2). This association remained significant after Bonferroni correction.

**Table 5:** Results of the linear regression analyses relating baseline UPSIT® score to comprehensive cognitive test scores at follow-up

	*N*		β	*B* (95% CI)	Sign. (*p*)
Rey’s Auditory Verbal Learning Test – delayed recall	62	Model 1	0.390	0.749 (0.292; 1.205)	0.002*
		Model 2	0.200	0.383 (−0.108; 0.874)	0.124
		Model 3	0.198	0.380 (−0.123; 0.883)	0.136
		Model 4	0.214	0.410 (−0.117; 0.937)	0.125
Stroop Color-Word test	61	Model 1	0.191	0.281 (−0.095; 0.657)	0.141
		Model 2	0.050	0.073 (−0.359; 0.505)	0.737
		Model 3	0.035	0.051 (−0.390; 0.493)	0.817
		Model 4	0.018	0.027 (−0.438; 0.492)	0.908
Forward Digit Span	62	Model 1	0.113	0.238 (−0.303; 0.778)	0.382
		Model 2	0.025	0.053 (−0.579; 0.686)	0.867
		Model 3	−0.004	−0.007 (−0.650; 0.635)	0.982
		Model 4	0.048	0.100 (−0.565; 0.766)	0.764
Reverse Digit Span	62	Model 1	0.284	0.479 (0.062; 0.895)	0.025*
		Model 2	0.120	0.202 (−0.272; 0.676)	0.396
		Model 3	0.087	0.146 (−0.333; 0.625)	0.543
		Model 4	0.134	0.226 (−0.270; 0.723)	0.336
Phonemic fluency	62	Model 1	0.302	0.524 (0.096; 0.951)	0.017*
		Model 2	0.277	0.481 (−0.017; 0.979)	0.058
		Model 3	0.236	0.410 (−0.090; 0.910)	0.106
		Model 4	0.341	0.593 (0.100; 1.086)	0.019*
Semantic fluency	61	Model 1	0.476	0.817 (0.427; 1.207)	< 0.001*
		Model 2	0.420	0.722 (0.262; 1.182)	0.003*
		Model 3	0.390	0.669 (0.204; 1.134)	0.006*
		Model 4	0.454	0.779 (0.302; 1.255)	0.002*

Model 1 for UPSIT® only

Model 2: adjusted for sex and age

Model 3: as model 2 with adjustment for disease duration

Model 4: as model 3 with adjustment for time between follow-up and baseline

β: standardized coefficient; B: unstandardized coefficient; *CI* confidence interval

*Significant (*p* < 0.05)

## Discussion

In this ten-year longitudinal cohort study in PD patients, worsening olfactory function was associated with a decline in global cognitive functioning, as measured with the MMSE, and not with changes in any of the other motor and other non-motor symptoms measured. Comprehensive cognitive testing in a subset of patients revealed that worsening olfactory function was specifically associated with a decrease in semantic fluency, but not in phonemic fluency or other cognitive functions. A post hoc analysis did not show that other clinical measures could explain these observed correlations (data not shown). Furthermore, lower baseline olfactory test score was associated with worse global cognitive functioning, more specifically semantic fluency, after ten years follow-up.

The results of the present longitudinal study confirm the observation in our earlier cross-sectional study of a correlation between olfactory function and global cognitive function. However, in our cross-sectional study we observed additional correlations between olfactory function and both motor and non-motor symptoms (depression, anxiety, autonomic dysfunction, and sleep disturbances) [24]. Cross-sectional data limit the ability to establish causality or assess time-related effects. On the other hand, participant dropouts might have influenced our longitudinal results, potentially explaining why we were unable to replicate all previous cross-sectional findings.

Our present observation that olfactory function is unrelated to motor symptom severity aligns with previous longitudinal studies [1, 50]. However, this contrasts with the study by He et al., which reported worse motor symptoms in hyposmic compared to normosmic PD patients [27]. One possible explanation for this discrepancy is the difference in follow-up duration. The relatively short follow-up in He et al.’s study may have led to an overestimation of the association, as early differences between hyposmic and normosmic patients could diminish over time.

To date, little research has been conducted on the relationship between olfaction and cognitive functioning in PD patients. Our results confirm the observation in the longitudinal study by He et al. that hyposmic PD patients had lower MMSE scores at baseline, indicating worse cognitive functioning, compared to normosmic PD patients [27]. The results of our study are also in line with previous data that focused on the prediction of cognitive decline using baseline olfactory testing. For example, one study showed that baseline olfactory function was related to change in cognitive functioning over time [29]. In another study olfactory dysfunction at baseline increased the subsequent risk of dementia in PD over a long period of time (median six years) [30].

In the subgroup of patients in which we performed comprehensive cognitive testing, we found that worsening of olfactory function was associated with a decrease in semantic fluency, but not in phonemic fluency. Successful performance in both semantic and phonemic fluency requires executive control to constantly monitor words in a specific category already named or letters already processed, as well to switch between letters [51]. While there is shared variance between performance on semantic and phonemic fluency tasks, these measures do capture distinct cognitive processes [52]. These distinct cognitive processes are expressed as a neural dissociation, with semantic fluency deficits more often occurring after damage to the left temporal regions [53–55], and phonemic fluency deficits more often seen after damage to the frontal regions [53, 55–57]. There is no consistent evidence on the role of white matter tracts in each fluency measure. An earlier longitudinal cohort study found that a decline in semantic fluency, not phonemic fluency, was predictive of later cognitive decline in PD [58]. The authors suggested that this reflects a more posterior and cortical degenerative process in PD associated with deficits in semantic fluency and likely driven by a non-dopaminergic etiology. Given the observed link between worsening olfactory function and semantic fluency decline, our findings may suggest that olfactory dysfunction in PD is indicative of neurodegenerative processes affecting temporal regions rather than frontal areas.

Olfactory dysfunction in PD is thought to result from α-synuclein pathology affecting both the olfactory bulb and central olfactory processing areas, including the piriform cortex, amygdala, and orbitofrontal cortex. Additionally, disruptions in specific neurotransmitter systems, particularly cholinergic pathways, may further contribute to, or even precede, olfactory impairment [59]. Given that cognitive decline in PD is linked to neurodegeneration in overlapping brain regions, such as orbitofrontal cortex and temporal lobe, these shared mechanisms could explain the observed association between olfactory and cognitive impairment. Furthermore, the ongoing decline in olfactory function may reflect the spread of disease-specific pathology to cortical brain regions as the disease progresses, in addition to previously recognized changes in the olfactory bulb [32].

The fact that baseline olfactory function is associated with later cognitive functioning in the present and previous studies [29–31], suggests that testing olfaction in an early stage of disease may help predict imminent cognitive decline. From that perspective, it is a major advantage that olfactory tests are easy to administer, non-invasive, and unaffected by the use of dopaminergic medication or motor functioning. While not a standalone diagnostic tool, olfactory testing may contribute to identifying patients at higher risk for cognitive decline in PD when used alongside other biomarkers, as has been done for Alzheimer’s disease [60]. Obviously, this hypothesis should be addressed in larger patient populations using a prospective longitudinal study design and multimodal testing.

An important strength of our study is the length of the follow-up period, averaging ten years. This extended duration allowed us to track the progression of olfactory and cognitive decline over the course of many years, providing valuable insights into their longitudinal relationship in PD. Moreover, it enabled the identification of long-term patterns that may not be detectable in shorter studies, strengthening the reliability of our findings on disease progression. Additionally, both baseline and follow-up assessments were carried out by a single neurologist specialized in movement disorders (DR). This consistency reduces the risk of interrater variability and helps ensure that participants met clinical criteria for PD throughout the study. Another strength of our study is the use of a well-validated self-administered odor identification test that has been widely employed in PD research [59, 61]. This highly reliable test (test–retest *r* = 0.92) correlates well with other types of olfactory tests, most notably odor detection threshold tests (e.g., Spearman *r* = 0.65 in a study of 733 subjects) [62]. However, unlike threshold tests, self-administered tests such as the UPSIT® preclude potential examiner influences on the test measure.

Our study has several limitations. First, since some patients declined participation in the comprehensive cognitive testing due to its burdensomeness, a sampling bias may have occurred. Thus, many patients who did not participate in this testing had lower MMSE scores. As a result, the correlation we found with semantic fluency could have been attenuated and the detection of other potential correlations missed. Second, our cohort was relatively young at baseline, which may partially explain the modest cognitive decline observed over ten years. Third, to reduce the total duration of study visits, we conducted a limited neuropsychological assessment, using a single test per cognitive domain. Since the official criteria for diagnosing mild cognitive impairment (MCI) and PD dementia (PDD) recommend at least two tests per domain, our approach may have underestimated cognitive impairment and its associations with other variables [63, 64]. Fourth, since the UPSIT® was completed unsupervised at baseline, olfactory function may have been underestimated at that time. As a result, the actual decline in olfactory function over ten years may have been larger than what we measured. This, in turn, suggests that the observed relationship between olfactory function and cognition, particularly semantic fluency, might also have been stronger than reported, although, in general, self-administration of the UPSIT® does not differ from proctored administration [65]. Finally, it would have been of interest to also have measured thresholds in addition to odor identification test scores to better define the relationship between olfaction and semantic fluency.

In conclusion, we found that over a ten-year period the decline in olfactory function in PD was associated with worsening cognitive function, particularly semantic fluency. While olfactory testing alone most likely is not sufficient for predicting cognitive decline, our observations suggest that it may help identify patients at an increased risk when used alongside other biomarkers. This could be particularly relevant in research settings or for refining patient stratification in future clinical trials.

## Supplementary Information

Below is the link to the electronic supplementary material.

Supplementary file1 (DOCX 36 KB)

## Data Availability

The datasets from this clinical research are available from the corresponding author upon reasonable request.
