# Supplementary material for: Olfactory function is longitudinally associated with semantic fluency in Parkinson’s disease: a cohort study
Source: J Neurol. 2025 Sep 2;272(9):604. doi: 10.1007/s00415-025-13337-0 (PMC12405024; doi:10.1007/s00415-025-13337-0)
Supplement: Supplementary file 1 — Supplementary file1 (DOCX 36 KB) [file 415_2025_13337_MOESM1_ESM.docx]

**Ref.: JOON-D-25-01798R2**

**Supplementary material**

Title: Olfactory function is longitudinally associated with semantic fluency in Parkinson’s disease: a cohort study; *Journal of Neurology*

Authors: Dareia S. Roos, MD; Henk W. Berendse, MD, PhD; Richard L. Doty, PhD; Jos WR Twisk, PhD; Martin Klein, PhD

Corresponding author: DS Roos

-Amsterdam UMC location Vrije Universiteit Amsterdam, Department of Neurology, de Boelelaan 1117, Amsterdam, The Netherlands

-Amsterdam Neuroscience, Neurodegeneration, Amsterdam, The Netherlands

De Boelelaan 1117, 1081 HV, Amsterdam

[d.roos@amsterdamumc.nl](mailto:d.roos@amsterdamumc.nl)

T: 0031(0)20-7323799

ORCID 0000-0001-7961-1768

**Supplement**

Table 1. Patient characteristics at baseline of patients included in the follow-up study compared to the patients that declined to participate or were lost to follow-up.

|  | Patients included in study  (n = 90) | Patients not included in study  (n = 205) | Sign. (*p*) |
| --- | --- | --- | --- |
| Age, mean (SD) [range], y | 58.3 (9.4) [27-74] | 68.3 (9.2) [45-89] | <0.001* |
| Males % (n) | 68.9 (62) | 57.1 (117) | 0.070 |
| Disease duration, mean (SD) [range], y | 4.5 (5.2) [0-34] | 5.6 (4.9) [0-33] | 0.005* |
| LEDD, mean (SD) [range], mg | 227.4 (352) [0-1255] | 362.6 (482) [0-3645] | 0.011* |
| UPSIT® score, mean (SD) [range] | 22.2 (7.6) [3-36] | 19.5 (7.1) [5-37] | 0.003* |
| MMSE score, mean (SD) [range] | 28.6 (1.5) [23-30] | 26.7 (3.7) [7-30] | <0.001* |

LEDD: levodopa equivalent daily dosage; MMSE: Mini-Mental State Examination; *Significant (*p*<0.05).

**Supplement**

Table 2. Baseline characteristics for the groups of patients with and without comprehensive cognitive testing at follow-up.

|  | Patients with comprehensive cognitive testing (n = 62) | Patients without comprehensive cognitive testing (n=28) | Sign. (*p*) |
| --- | --- | --- | --- |
| Age, mean (SD) [range], y | 55.9 (9.4) [27-74] | 63.7 (7.1) [53-74] | <0.001* |
| Males % (n) | 75.8 (47) | 53.6 (15) | 0.049* |
| Disease duration, mean (SD) [range], y | 4.5 (5.4) [0-34] | 4.6 (4.8) [1-17] | 0.971 |
| LEDD, mean (SD) [range], mg | 173 (294) [0-1140] | 347 (439) [0-1255] | 0.104* |
| UPSIT® score, mean (SD) [range] | 23.6 (7.1) [9-36] | 19.1 (8.0) [3-32] | 0.010* |
| MMSE score, mean (SD) [range] | 28.8 (1.2) [24-20] | 28.1 (2.0) [23-30] | 0.036* |

LEDD: levodopa equivalent daily dosage; MMSE: Mini-Mental State Examination; *Significant (*p*<0.05).

**Supplement**

Table 3. Change in test scores between baseline and follow-up.

|  | Baseline | | Follow-up | |  | Change | |
| --- | --- | --- | --- | --- | --- | --- | --- |
|  | N | Mean (SD) [range] | N | Mean (SD) [range] | Sign. | N | Mean (SD) |
| UPDRS III | 88 | 21.7 (10.6) [4-46] | 89 | 25.6 (11.9) [6-66] | 0.01 | 87 | 3.5 (12.3) |
| MMSE | 88 | 28.6 (1.5) [23-30] | 87 | 27.1 (3.6) [8-30] | <0.001 | 85 | -1.5 (3.1) |
| SCOPA-SLEEP | 84 | 11.1 (7.9) [2-35] | 89 | 12.8 (7.8) [1-37] | 0.108 | 82 | 0.8 (8.8) |
| SCOPA-AUT | 86 | 11.0 (7.2) [0--33] | 88 | 16.1 (7.5) [1-37] | <0.001 | 84 | 4.7 (7.2) |
| SCOPA-PC | 87 | 0.8 (1.1) [0-5] | 89 | 1.8 (1.9) [0-9] | <0.001 | 86 | 1.0 (1.9) |
| BAI | 87 | 11.9 (9.2) [0-41] | 89 | 12.7 (11.3) [0-37] | 0.498 | 86 | 0.9 (12.8) |
| BDI | 89 | 8.8 (7.4) [0-44] | 89 | 7.4 (5.8) [0-38] | 0.062 | 88 | -1.4 (6.8) |
|  |  |  |  |  |  |  |  |
| Rey’s Auditory Verbal Learning Test – delayed recall | 75 | 44.5 (10.4) [19-71] | 62 | 35.5 (13.6) [13-67] | <0.001 | 51 | -8.2 (12.0) |
| Stroop Color-Word test | 70 | 52.2 (8.6) [30-71] | 61 | 50.9 (10.5) [24-76] | 0.521 | 50 | -0.9 (9.4) |
| Forward Digit Span | 75 | 51.7 (11.8) [23-78] | 62 | 55.6 (15.0) [20-87] | 0.031 | 52 | 4.3 (13.8) |
| Reverse Digit Span | 75 | 54.6 (10.2) [33-86] | 62 | 54.2 (12.0) [36-104] | 0.785 | 52 | 0.4 (11.6) |
| Phonemic fluency | 72 | 51.5 (10.9) [27-79] | 62 | 46.3 (12.3) [21-85] | 0.01 | 50 | -3.9 (10.5) |
| Semantic fluency | 71 | 52.6 (9.4) [35-79] | 62 | 41.9 (12.2) [14-79] | <0.001 | 50 | -10.8 (11.8) |

UPDRS III: Unified Parkinson’s Disease Rating Scale motor subscale; MMSE: Mini-Mental State Examination; SCOPA: SCales for Outcomes in PArkinson’s Disease; AUT: autonomic dysfunction; PC: psychiatric complications; BAI: Beck Anxiety Inventory; BDI: Beck Depression Inventory.
